# Platform-Independent Genome-Wide Pattern of DNA Copy-Number Alterations Predicting Astrocytoma Survival and Response to Treatment Revealed by the GSVD Formulated as a Comparative Spectral Decomposition

**DOI:** 10.1371/journal.pone.0164546

**Published:** 2016-10-31

**Authors:** Katherine A. Aiello, Orly Alter

**Affiliations:** 1 Scientific Computing and Imaging Institute, University of Utah, Salt Lake City, Utah, United States of America; 2 Department of Bioengineering, University of Utah, Salt Lake City, Utah, United States of America; 3 Huntsman Cancer Institute, University of Utah, Salt Lake City, Utah, United States of America; 4 Department of Human Genetics, University of Utah, Salt Lake City, Utah, United States of America; National Institute of Environmental Health Sciences, UNITED STATES

## Abstract

We use the generalized singular value decomposition (GSVD), formulated as a comparative spectral decomposition, to model patient-matched grades III and II, i.e., lower-grade astrocytoma (LGA) brain tumor and normal DNA copy-number profiles. A genome-wide tumor-exclusive pattern of DNA copy-number alterations (CNAs) is revealed, encompassed in that previously uncovered in glioblastoma (GBM), i.e., grade IV astrocytoma, where GBM-specific CNAs encode for enhanced opportunities for transformation and proliferation via growth and developmental signaling pathways in GBM relative to LGA. The GSVD separates the LGA pattern from other sources of biological and experimental variation, common to both, or exclusive to one of the tumor and normal datasets. We find, first, and computationally validate, that the LGA pattern is correlated with a patient’s survival and response to treatment. Second, the GBM pattern identifies among the LGA patients a subtype, statistically indistinguishable from that among the GBM patients, where the CNA genotype is correlated with an approximately one-year survival phenotype. Third, cross-platform classification of the Affymetrix-measured LGA and GBM profiles by using the Agilent-derived GBM pattern shows that the GBM pattern is a platform-independent predictor of astrocytoma outcome. Statistically, the pattern is a better predictor (corresponding to greater median survival time difference, proportional hazard ratio, and concordance index) than the patient’s age and the tumor’s grade, which are the best indicators of astrocytoma currently in clinical use, and laboratory tests. The pattern is also statistically independent of these indicators, and, combined with either one, is an even better predictor of astrocytoma outcome. Recurring DNA CNAs have been observed in astrocytoma tumors’ genomes for decades, however, copy-number subtypes that are predictive of patients’ outcomes were not identified before. This is despite the growing number of datasets recording different aspects of the disease, and due to an existing fundamental need for mathematical frameworks that can simultaneously find similarities and dissimilarities across the datasets. This illustrates the ability of comparative spectral decompositions to find what other methods miss.

## Introduction

Recurring DNA copy-number alterations (CNAs) have been recognized as a hallmark of cancer for >100 years [1–3], yet what these alterations imply about a solid tumor’s development and progression, and a patient’s diagnosis, prognosis, and treatment remains poorly understood. This is despite the growing number of high-dimensional datasets, recording different aspects of a single disease, such as DNA copy-number profiles of two or more cell types from the same set of patients, possibly measured more than once by different platforms. This is due to an existing fundamental need for mathematical frameworks that can create a single coherent model from, i.e., simultaneously find similarities and dissimilarities across such datasets, arranged in two or more tables, of two or possibly more dimensions, i.e., matrices or tensors, of matched columns but independent rows.

A recent comparison of DNA copy-number profiles of primary tumor and normal cells from the same set of ovarian serous cystadenocarcinoma (OV) patients, measured by the same set of platforms, uncovered three tumor-exclusive platform-consistent chromosome arm-wide patterns of DNA CNAs that are correlated with a patient’s survival and response to platinum therapy [4]. The datasets had been publicly available in the Cancer Genome Atlas (TCGA) since 2011, and analyzed by using several methods [5]. The patterns, however, remained unknown until the datasets were modeled in 2015 by using a novel comparative spectral decomposition, the tensor generalized singular value decomposition (GSVD). For >30 years prior, statistically the best indicator of OV survival was the tumor’s stage at diagnosis [6]. About 25% of primary OV tumors are resistant to platinum therapy, the first-line treatment, yet no diagnostic existed to distinguish resistant from sensitive tumors before the treatment [7].

A previous comparison of copy-number profiles of primary tumor and normal cells from the same set of glioblastoma (GBM) brain cancer patients, uncovered a tumor-exclusive genome-wide pattern of CNAs that is correlated with a patient’s survival and response to chemotherapy [8]. The datasets had been publicly available in TCGA since 2008 [9]. The pattern, however, remained unknown until the datasets were modeled in 2012 by using the GSVD [10–16], formulated as a comparative spectral decomposition [17] (see also [18–30]). For >50 years prior, statistically the best indicator of GBM outcome was the patient’s age at diagnosis [31–33] (see also [34, 35]). Copy-number subtypes of GBM, i.e., grade IV astrocytoma, which are predictive of survival and response to treatment were not conclusively identified [36, 37].

## Results

### GSVD Comparison of Patient-Matched LGA Brain Tumor and Normal DNA Copy-Number Profiles

**Fig 1:**
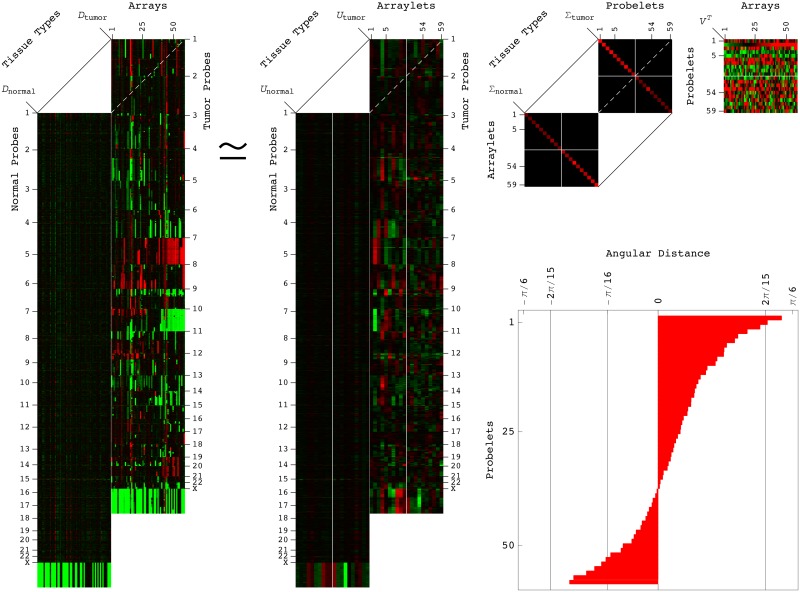
GSVD of the patient-matched LGA tumor and normal DNA copy-number profiles. The structure of the LGA discovery, tumor and normal datasets *D_i_* is that of two matrices of 59 matched columns (i.e., patients), and 933,827, not necessarily matched or equal in numbers, rows (i.e., tumor and normal genomic regions, or Affymetrix probes). The GSVD of Eq (1) simultaneously separates the datasets into a single set of normalized, not necessarily orthogonal probelets *V^T^* (i.e., patterns of variation across the patients), which are identical for both datasets, but correspond to different sets of generalized singular values Σ_*i*_ (i.e., weights, or superposition coefficients) and orthonormal arraylets *U_i_* (i.e., patterns of variation across the genome) in each dataset. The GSVD is depicted in a raster display, with relative DNA copy-number gain (red), no change (black), and loss (green), which explicitly shows only the first through the 10th, and the 50th through the 59th probelets and corresponding tumor and normal arraylets, and tumor and normal generalized singular values. The angular distances of Eq (4) define the significance of each probelet in the tumor dataset relative to its significance in the normal dataset in terms of the ratio of the corresponding tumor to normal generalized singular values [17]. The inset bar chart shows that the angular distances largest in magnitude correspond to the first and second probelets, and are > 2*π*/15, whereas the magnitude of the angular distance that corresponds to the 53rd probelet is < *π*/16.

To identify CNAs that might predict grades III and II, i.e., lower-grade astrocytoma (LGA) patients’ outcomes, we, therefore, used the GSVD to model TCGA patient-matched LGA tumor and normal DNA copy-number profiles [38]. We selected patient-matched Affymetrix-measured DNA copy-number profiles of primary LGA tumor and normal tissue samples from a discovery set of 59 patients (Methods and S1 Dataset). The structure of these tumor and normal datasets is that of two full column-rank matrices $D_{1}\in R^{M_{1}\times N}$ and $D_{2}\in R^{M_{2}\times N}$ of *N* = 59 matched columns (i.e., patients), but independent, i.e., not necessarily matched or equal in numbers *M*_1_, *M*_2_ = 933,827 rows (i.e., tumor and normal genomic regions, or Affymetrix probes), where *M*_1_, *M*_2_≫*N* (Fig 1).

The GSVD simultaneously separates the two matrices, or tumor- and normal-specific datasets, into paired weighted sums of outer products, of each normalized, not necessarily orthogonal right basis vector, or “probelet” $v_{n}^{T}$ (i.e., a pattern of variation across the patients), which is identical for both datasets, combined with one of the two corresponding orthonormal left basis vectors, or “tumor arraylet” *u*_1,*n*_ and “normal arraylet” *u*_2,*n*_ (i.e., the tumor- and normal-specific patterns of variation across the genome),

$$D_{i}=U_{i}\Sigma_{i}V^{T}=\sum_{n=1}^{N}\sigma_{i,n}u_{i,n}⊗v_{n}^{T},\;i=1,2.$$ (1)

The significance of a probelet $v_{n}^{T}$ in either the tumor dataset *D*_1_ or the normal dataset *D*_2_, in terms of the “generalized fraction” of the overall information that it captures in the dataset, is proportional to the corresponding nonnegative generalized singular value *σ*_1,*n*_ or *σ*_2,*n*_, respectively,

$$p_{i,n}=\sigma_{i,n}^{2}/\sum_{n=1}^{N}\sigma_{i,n}^{2},\;i=1,2.$$ (2)

The “generalized normalized Shannon entropy” is defined to measure the complexity of each dataset in terms of the distribution of the overall information in the dataset among the probelets,

$$0\leq d_{i}=-{\left(\log N\right)}^{-1}\sum_{n=1}^{N}p_{i,n}\log p_{i,n}\leq 1,\;i=1,2.$$ (3)

An entropy of zero corresponds to an ordered and redundant dataset, in which all the information is captured by a single probelet. An entropy of one corresponds to a disordered and random dataset, in which all probelets are of equal significance.

Following the relation of the GSVD to the cosine-sine (CS) decomposition [14], the significance of a probelet $v_{n}^{T}$ in the tumor dataset *D*_1_ relative to its significance in the normal dataset *D*_2_ is defined by the “angular distance” *θ*_*n*_ [17],

$$-\pi /4\leq \theta_{n}=\arctan\left(\sigma_{1,n}/\sigma_{2,n}\right)-\pi /4\leq \pi /4.$$ (4)

Probelets for which *θ*_*n*_ ∼ ±*π*/4 are exclusive to either the tumor or the normal dataset, respectively, whereas probelets for which |*θ*_*n*_|∼0 are common to both. The probelets are arranged in decreasing order of their angular distances, i.e., their significance in the tumor relative to the normal dataset. The GSVD is unique, except in degenerate subspaces, defined by subsets of equal pairs of generalized singular values *σ*_1,*n*_ and *σ*_2,*n*_, and up to phase factors of ±1 of each probelet $v_{n}^{T}$ and the corresponding tumor and normal arraylets *u*_1,*n*_ and *u*_2,*n*_.

We find that the two most tumor-exclusive patterns of variation across the patients, i.e., the first and second probelets, with angular distances *θ*_1_, *θ*_2_ > 2*π*/15, are also the first and third most significant probelets in the tumor dataset, with >8% and 5% of the information in this dataset, respectively (Fig A in S1 Appendix). The 53rd probelet, which with ∼10% of the information is the most significant probelet in the normal dataset, is approximately common to both datasets with |*θ*_53_| < *π*/16.

The GSVD, therefore, creates a single coherent model of the two datasets by simultaneously identifying unique probelets that are significant in, and common to the two datasets, as well as those that are significant in, and exclusive to either one of the datasets. We interpret the model accordingly, in terms of the biological and experimental phenomena that are common to the LGA tumor and normal profiles, as well as those that are exclusive to the LGA tumor or the normal profiles.

#### The GSVD Reveals a Genome-Wide LGA Tumor-Exclusive Pattern of CNAs Encompassed in the GBM Pattern

**Fig 2:**
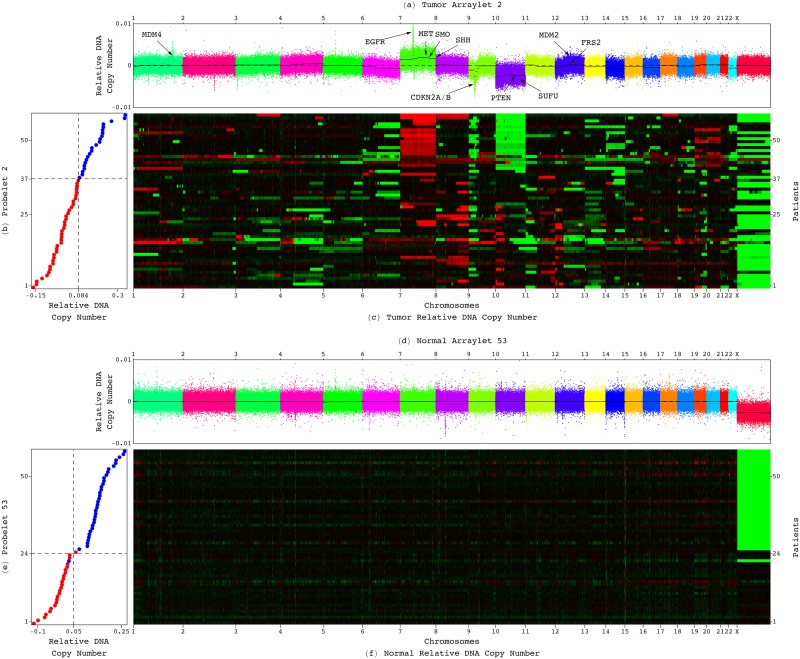
Significant probelets and corresponding tumor and normal arraylets revealed by the GSVD of the LGA discovery datasets. (*a*) Plot of the second most LGA tumor-exclusive tumor arraylet describes a genome-wide pattern of co-occurring CNAs across 933,827 Affymetrix probes. The probes are ordered, and their copy numbers are colored, according to each probe’s chromosomal location. This LGA pattern is encompassed in a GBM pattern, which was previously uncovered by the GSVD [8]. Segments (black lines) that were identified in the GBM pattern, and are amplified or deleted in the LGA pattern, are also amplified or deleted in the GBM pattern, respectively, and at a greater magnitude (Fig 3). The GBM-associated LGA-shared focal CNAs (black) include, e.g., a gain of a segment on chromosome 1 containing *MDM4*. (*b*) Plot of the second LGA probelet describes the variation of the weight, or superposition coefficient of the LGA pattern in the tumor profiles of the 59 patients. The second probelet classifies the patients into two groups of low (red) and high (blue) weights, which are of statistically significantly different prognoses (Fig 4). (*c*) Raster display of the tumor dataset shows the correspondence between the tumor profiles and the second LGA probelet and tumor arraylet. (*d*) Plot of the 53rd LGA normal arraylet, which is the most significant in the normal dataset, describes a deletion of the X chromosome. (*e*) Plot of the 53rd LGA probelet, which is approximately common to the tumor and normal datasets, describes a classification of the patients by gender into females (red) and males (blue). The corresponding hypergeometric *P*-value is <10^−13^. (*f*) Raster display of the normal dataset shows the male-specific X chromosome deletion across the normal genomes. This biological variation is conserved in the patient-matched LGA tumor genomes. The GSVD separates this variation from the second LGA tumor arraylet.

**Fig 3:**
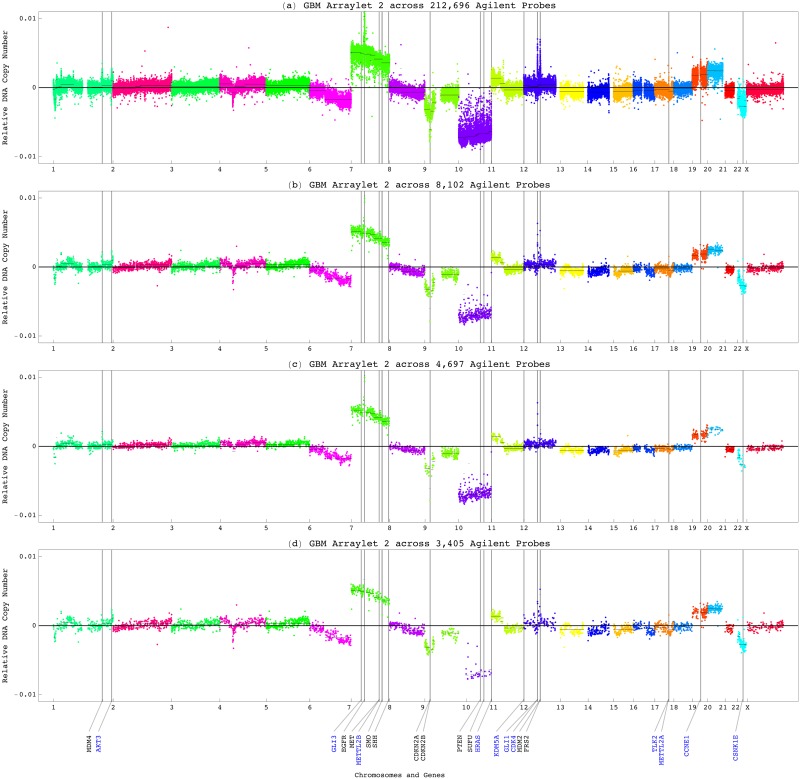
GBM genome-wide pattern of co-occurring CNAs previously uncovered by the GSVD of GBM tumor and normal profiles. (*a*) Plot of the second most GBM tumor-exclusive tumor arraylet, which was previously uncovered by the GSVD [8], describes a genome-wide pattern of co-occurring CNAs across 212,696 Agilent probes. The GBM pattern, which encompasses the LGA pattern (Fig 2), consists of LGA-shared (black) and GBM-specific (blue) CNAs, including, e.g., gains of segments on chromosome 1 containing *MDM4* and *AKT3*, respectively. (*b*) Both LGA-shared and GBM-specific CNAs are visible across the 8,102 Affymetrix-matched Agilent probes, even though these are <4% of the probes that constitute the GBM pattern. (*c*) The LGA-shared CNAs, e.g., in *MDM4*, are visible across the 4,697 Affymetrix-matched consistently-aberrated Agilent probes. (*d*) The GBM-specific CNAs, e.g., in *AKT3*, are visible across the 3,405 remaining probes.

In a previous GSVD comparison of patient-matched Agilent-measured DNA copy-number profiles of primary GBM tumor and normal samples, we found that the second most GBM tumor-exclusive tumor arraylet describes a genome-wide pattern of co-occurring CNAs that is correlated with a GBM patient’s outcome [8]. Now, we find that the second LGA tumor arraylet describes a genome-wide pattern of co-occurring CNAs across the Affymetrix probes, which is similar to the GBM pattern (Figs 2 and 3, and Fig B in S1 Appendix). To compare the LGA to the GBM pattern, we assigned to the LGA pattern CNAs in the chromosomes and chromosome arms as well as the genomic segments that were identified in the GBM pattern (S2 Dataset). We find that the LGA pattern is encompassed in the GBM pattern. Chromosomes, chromosome arms, and segments that are amplified or deleted in the LGA pattern are also amplified or deleted in the GBM pattern, respectively, and at a greater magnitude; some of those that show no copy-number change in the LGA pattern are amplified or deleted in the GBM pattern.

Dominant in the LGA pattern, but at a lesser magnitude than in the GBM pattern, are the known, GBM-associated gain of chromosome 7 and loss of chromosome 10 [36, 37]. Also dominant in the LGA pattern, also at a lesser magnitude than in the GBM pattern, are GBM-associated focal CNAs [8] (see also [9, 39]). Among these, we find amplifications and deletions that contribute to decreased activity of the tumor suppressor protein p53. These include gains of segments containing the p53-inactivating protein-encoding *MDM4* (1q32.1) and the p53-degrading protein-encoding *MDM2* (12q15), and losses of segments containing *CDKN2A* and *CDKN2B* (9p21.3), and *PTEN* (10q23.31). The tumor suppressor protein encoded by *PTEN* negatively regulates the Mdm2 protein via the Akt pathway. Of the three known transcript variants of *CDKN2A*, one encodes p14^ARF^, which is a p53-stabilizing, Mdm2-sequestering protein. The other two variants encode isoforms of the tumor suppressor protein p16^INK4A^. *CDKN2B* encodes for the transforming growth factor-*β* (TGF-*β*) -induced growth inhibitor p15^INK4B^ [40]. Together with the retinoblastoma (Rb) protein tumor suppressor, and in parallel to p53 and p14^ARF^, p16^INK4A^ and p15^INK4B^ act at a checkpoint for human normal to tumor cell transformation, promoting cell cycle arrest, apoptosis, and senescence in response to rat sarcoma virus (Ras) -mediated hyperactive growth factor signaling [41–44]. Amplifications that are involved in increased growth factor signaling among the GBM-associated LGA-shared CNAs include gains of segments containing the epidermal growth factor receptor *EGFR* (7p11.2), the hepatocyte growth factor receptor *MET* (7q31.2), and the fibroblast growth factor receptor (FGFR) substrate *FRS2* (12q15) [45] (Fig C in S1 Appendix).

Additional LGA- and GBM-shared CNAs contribute to decreased activity of the tumor suppressor protein Ptch1, and increased downstream conversion of the oncogenes Gli1–3 into transcriptional activators by the Hedgehog (Hh) signaling pathway. These include gains of segments containing the Hh ligand-encoding *SHH* (7q36.3) and the Hh signal-transducing protein-encoding *SMO* (7q32.1), and a loss of a segment containing the Hh negative regulator protein-encoding *SUFU* (10q24.32) [46]. Note that reduced Ptch1 activity is also shared by the brain cancer medulloblastoma, where it was shown to contribute to the development of the tumor [47, 48] (Fig D in S1 Appendix).

The GBM pattern consists of additional CNAs that are missing from the LGA pattern, including the GBM-associated loss of the short arm of chromosome 9 (9p), and the long arm of chromosome 22 (22q). Among the GBM-specific CNAs we find amplifications that contribute to decreased Rb activity. These include gains of segments containing the viral protein-binding Rb region-interacting protein-encoding *KDM5A* (12p13.33) [49], the Rb-phosphorylating protein-encoding *CDK4* (12q14.1), and cyclin E1 *CCNE1* (19q12), which repression by Rb is necessary to prevent replication of senescent cells [50, 51]. Additional GBM-specific gains are of segments containing the oncogenes *AKT3* (1q44) [52] and Harvey Ras-encoding *HRAS* (11p15.5) [53]. We find, therefore, that the GBM-specific amplifications, of *AKT3*, *HRAS*, and genes involved in decreased Rb activity, together with the LGA-shared deletions of *CDKN2A* and *CDKN2B*, and CNAs involved in decreased activity of p53, enhance the opportunity for human normal to tumor cell transformation in response to growth factor signaling in GBM relative to LGA.

GBM-specific CNAs that contribute to increased conversion of the Gli oncogenes into transcriptional activators, include gains of segments containing the genes encoding for two of the three Gli proteins, *GLI3* (7p14.1) and *GLI1* (12q13.3), which was first identified in a screen of amplified DNA in a malignant human glioma tumor sample [54]. Also included is a loss of a segment containing the serine/threonine protein kinase-encoding *CSNK1E* (22q13.1). The encoded kinase CKI*ϵ* is one of two members of the casein kinase I (CKI) protein family that in the absence of Hh facilitate the conversion of the Gli proteins into transcriptional repressors [55]. These GBM-specific CNAs that are involved in increased levels of the Gli transcriptional activators, together with the LGA-shared CNAs that are involved in decreased activity of Ptch1, enhance the opportunity for proliferation in response to developmental signals in GBM relative to LGA [56].

Gains of segments containing putative drug targets are also among the GBM-specific CNAs, including the methyltransferases-encoding *METTL2B* (7q32.1) and *METTL2A* (17q23.2), and the serine/threonine kinase-encoding *TLK2* (17q23.2) [8, 57].

To additionally compare the LGA and GBM patterns, we identified 8,102 pairs of one-to-one overlapping Affymetrix and Agilent probes among the 933,827 Affymetrix probes of the LGA pattern and the 212,696 Agilent probes of the GBM pattern. Among these, we identified 4,697 pairs of one-to-one overlapping probes that are consistently aberrated in the LGA and GBM patterns. The LGA-shared CNAs in chromosomes, chromosome arms, and segments are visible in both the LGA and GBM patterns, across the 8,102, and, separately, the 4,697 pairs of probes, even though these are <1% and 4% of the probes that constitute the LGA and GBM patterns, respectively.

#### The GSVD Separates the LGA Pattern from CNVs Common to the Normal Human and LGA Tumor Genomes and Tumor-Exclusive Experimental Batch Effects

This is because the second tumor arraylet, which describes the LGA pattern, is mathematically orthogonal to the other tumor arraylets, which describe other sources of biological and experimental variation that compose the tumor dataset.

For example, the first tumor arraylet, which is mathematically the most significant arraylet in the tumor dataset, describes mostly unsegmented chromosomes [58, 59], each with a copy-number distribution that is approximately centered at the autosomal genome with a relatively large, chromosome-invariant width (Fig E in S1 Appendix and S3 Dataset). The first probelet, which is mathematically the most tumor-exclusive probelet, is correlated with a tumor-exclusive experimental variation in the hybridization plate of the LGA tumor samples, with both hypergeometric [60] and Mann-Whitney-Wilcoxon *P*-values <10^−2^ (Fig F in S1 Appendix). Together, the first probelet and tumor arraylet describe a tumor-exclusive experimental batch effect.

The 53rd normal arraylet, which is mathematically the most significant arraylet in the normal dataset, and the 53rd LGA tumor arraylet (Fig G in S1 Appendix), both describe a deletion of the X chromosome relative to the autosomal genome. Consistently, the 53rd probelet, which is mathematically approximately common to the tumor and normal datasets, classifies the patients by gender, with both hypergeometric and Mann-Whitney-Wilcoxon *P*-values <10^−9^. Together, the 53rd probelet and arraylets describe a male-specific X chromosome deletion, a CNV across the normal genomes that is conserved in the patient-matched LGA tumor genomes.

Note that although the male-specific X chromosome deletion is conserved in the tumor genomes, the LGA pattern, which is described by the second tumor arraylet, exhibits an unsegmented X chromosome copy-number distribution that is approximately centered at the autosomal genome with a relatively small, invariant width. This illustrates the separation of the LGA tumor-exclusive pattern from the male-specific X chromosome deletion that is common to the tumor and normal profiles.

This GSVD separation of the LGA tumor and normal datasets into probelets, and tumor and normal arraylets, is blind, that is, without a-priori knowledge of the sources of variation that compose the datasets. The TCGA annotations that describe the patients (e.g., gender), and the corresponding tumor and normal samples (e.g., the hybridization plate of the tumor vs. the normal samples), are used only to interpret the patterns of variation across the patients, and the tumor and normal genomes, which were uncovered by the GSVD.

#### The LGA Pattern is Correlated with LGA Outcome

**Fig 4:**
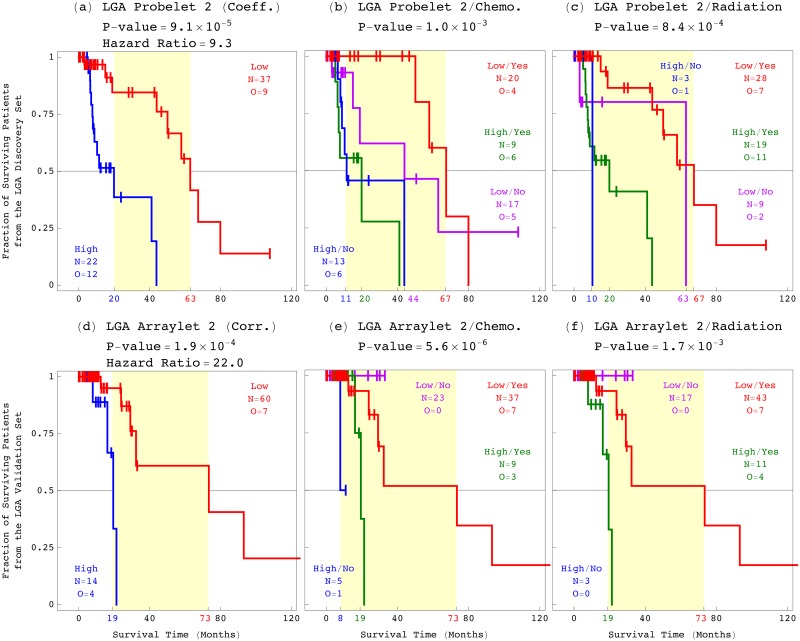
Survival analyses of the LGA patients classified by the LGA pattern and by treatment. (*a*) KM curves of the discovery set of 59 patients classified by the weights, or superposition coefficients of the LGA pattern in their tumor profiles, as listed in the second probelet (Fig 3). The 63-month KM median survival time of the group of patients with low coefficients is >3 times greater than that of the group of patients with high coefficients, with the corresponding log-rank test *P*-value <10^−4^. The univariate Cox proportional hazard ratio is >9. (*b*) Among the 29 patients in the discovery set treated by chemotherapy, the median survival time of the patients with low coefficients is ∼3.5 times greater than that of the patients with high coefficients. (*c*) Among the patients treated by radiation, the median survival times of patients with low and high coefficients are the same as among the chemotherapy-treated patients. (*d*) KM curves of the validation set of 74 patients classified by the Pearson correlation of the LGA pattern with their tumor profiles. The 73-month median survival time of the patients with low correlations is >3.5 times greater than that of the patients with high correlations, consistent with the median survival times of the patients in the discovery set. (*e*) The median survival times of the 46 chemotherapy-treated validation patients with low and high correlations are the same as those of the 74 validation patients, and consistent with those of the 27 chemotherapy-treated discovery patients. (*f*) The median survival times of the radiation-treated validation patients are the same as those of the validation patients, and consistent with those of the radiation-treated discovery patients.

To examine the correlation of the LGA pattern with an LGA patient’s survival, we classified the discovery set of patients based upon the weight of the pattern, that is, the superposition coefficient of the second LGA tumor arraylet, in each patient’s tumor profile. These coefficients are linearly proportional to the relative copy numbers listed in the second LGA probelet. For the cutoff to be consistent with that previously established for the GBM pattern [8], we scaled the second GBM arraylet correlation cutoff of 0.15 by the Euclidean-, i.e., 2-norm of the Pearson correlations of the discovery tumor profiles with the second LGA tumor arraylet. The second probelet classifies the discovery set of patients into two groups of statistically significantly different prognoses (Fig 4). The univariate Cox [61] proportional hazard ratio is >9. This means that a high weight of the LGA pattern in an LGA tumor’s profile confers >9 times the hazard of a low weight.

To examine the correlation of the pattern with response to treatment, we classified the discovery set of patients by the GSVD and, in addition, by chemotherapy or radiation. Among the patients who were treated by either chemotherapy or radiation, the Kaplan-Meier (KM) [62] median survival time of the groups of patients with low coefficients is ∼3.5 times, and ∼4 years greater than the median survival time of the groups of patients with high coefficients. A low weight of the LGA pattern in an LGA tumor’s profile is, therefore, correlated with a significantly longer survival time, also in response to chemotherapy or radiation.

To computationally validate that the LGA pattern is correlated with LGA outcome, we classified the Affymetrix-measured primary LGA tumor profiles of a validation set of 74 TCGA patients, mutually exclusive of the discovery set (S4 Dataset). The classification is based upon the correlation of the second LGA tumor arraylet with each patient’s tumor profile across the 933,827 Affymetrix probes. We find that the results of the survival analyses of the LGA validation set are consistent with those of the LGA discovery set. Note also that in classifying the tumor profiles, the 8,102 Agilent-matched Affymetrix probes and, separately, the 4,697 consistently-aberrated probes among them, give qualitatively the same and quantitatively similar results as the 933,827 Affymetrix probes.

### The GBM Pattern Identifies among the LGA Patients a Subtype, Similar to that among the GBM Patients, where the CNA Genotype is Correlated with an Approximately One-Year Survival Phenotype

**Fig 5:**
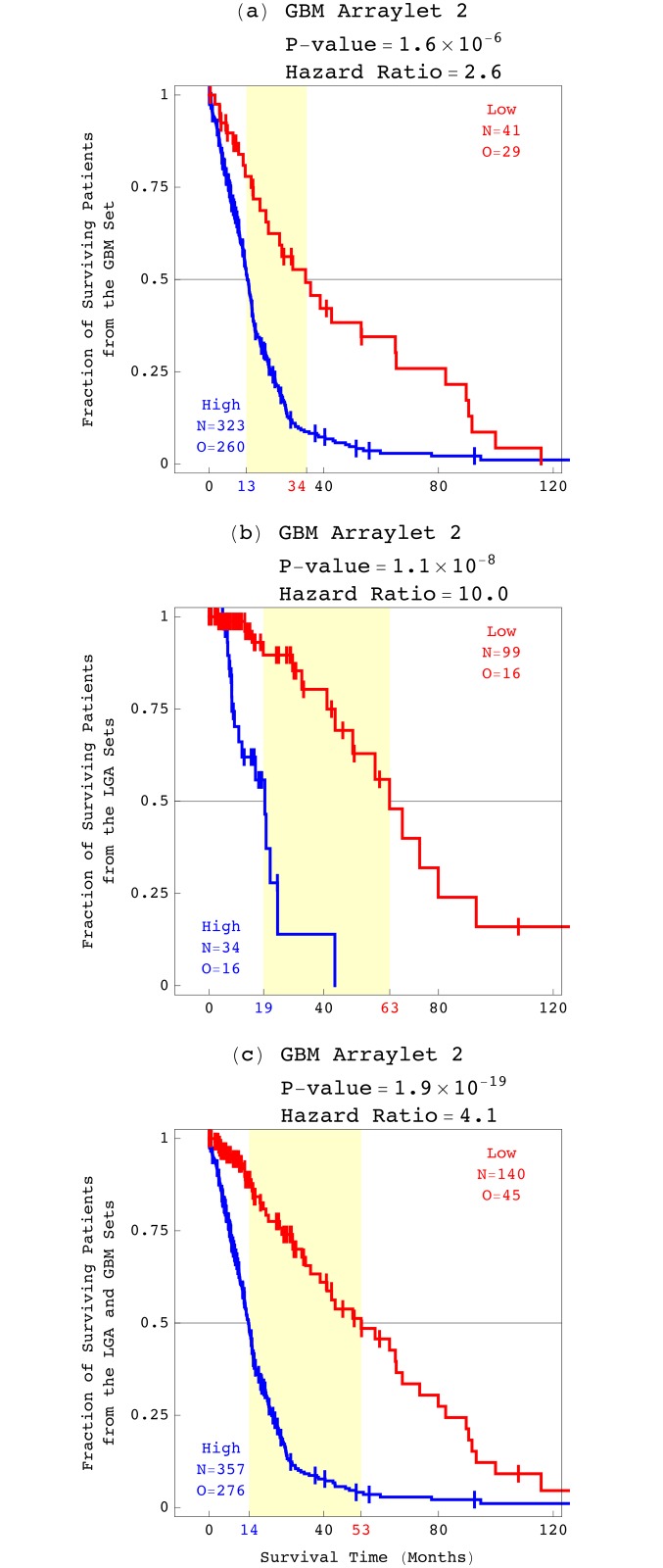
Survival analyses of the LGA and GBM patients classified by the GBM pattern. KM curves, log-rank test *P*-values, and Cox proportional hazard ratios of (*a*) the GBM set of 364 patients, (*b*) the LGA discovery and validation sets of 133 patients, and (*c*) the LGA and GBM sets of 497 patients.

Because the GBM pattern encompasses the LGA pattern, we also examined the correlation of the GBM pattern with an LGA patient’s survival. To start, we used the GBM pattern to classify the primary GBM tumor profiles of a set of 364 TCGA patients (S5 Dataset). We find that the GBM pattern is a platform-independent predictor of GBM survival. Classifying the GBM patients based upon the Affymetrix-measured tumor profiles, and across just the 4,697 probes (Fig 5), gives qualitatively the same and quantitatively similar results as the previous classification based upon the Agilent-measured profiles, across the 212,696 Agilent probes [8]. As in the previous classification, the KM median survival time of the group of patients with low correlations is >2.5 times, and >1.5 years greater than the approximately one-year median survival time of the group of patients with high correlations.

Next, we used the GBM pattern to classify the Affymetrix-measured tumor profiles of the 133 TCGA patients in the LGA discovery and validation sets. The survival analysis results are consistent with those based upon the correlation with the Affymetrix-derived LGA pattern across the 933,827 Affymetrix probes.

Because a high weight of the GBM pattern in either an LGA or a GBM tumor’s profile confers a greater hazard and a shorter survival time, we compared the survival of the groups of LGA and GBM patients that are identified by the GBM pattern. We find that the KM curves for these two groups overlap, with the corresponding log-rank test *P*-value >0.05, which means that the two groups are statistically indistinguishable based upon survival.

Classifying the 133 LGA and 364 GBM, i.e., 497 astrocytoma patients, based upon the weight of the GBM pattern in each patient’s tumor profile, we find that the GBM pattern is a predictor of survival among the general primary astrocytoma population, independent of grade, where the CNA genotype that the GBM pattern describes is correlated with an approximately one-year survival phenotype. We also assessed the distribution of several TCGA annotations of intratumor heterogeneity in each astrocytoma grade, including the tumor sample’s volume, the slide’s percents of tumor cells and nuclei, the portion’s weight, and the analyte’s and aliquot’s native, unamplified DNA quantities. We find that at the TCGA ranges for these annotations, the GBM pattern is independent of intratumor heterogeneity.

### The GBM Pattern is a Platform-Independent Predictor of Astrocytoma Outcome, Statistically Better Than, and Independent of Age, Grade, and Existing Laboratory Tests

**Fig 6:**
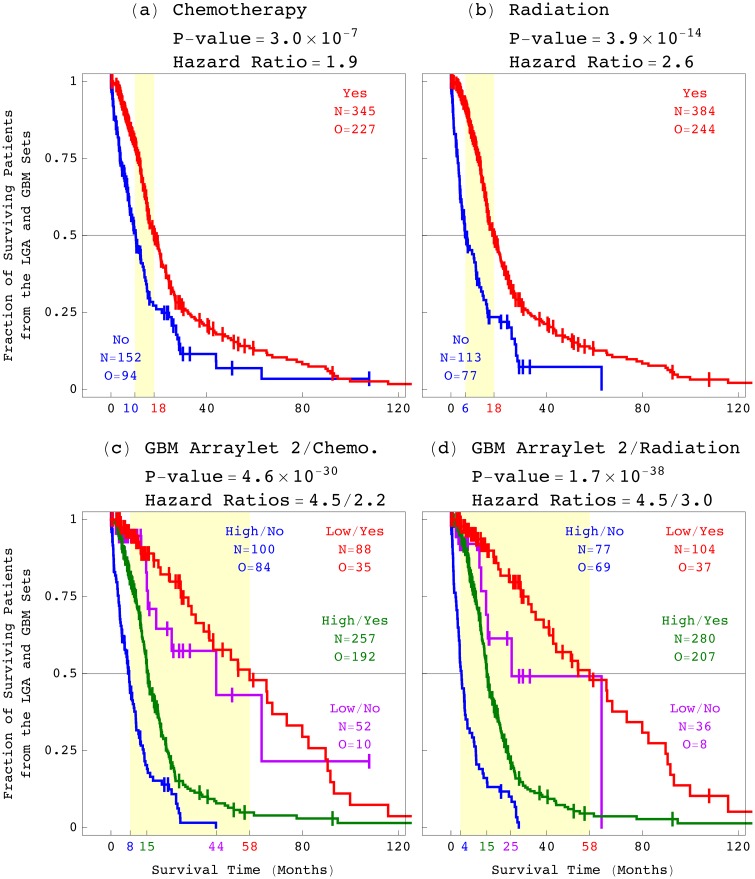
Survival analyses of the astrocytoma patients classified by treatment and by the GBM pattern. KM curves, log-rank test *P*-values, and Cox proportional hazard ratios of the 497 astrocytoma patients classified by (*a*) chemotherapy, (*b*) radiation, (*c*) the GBM pattern combined with chemotherapy, and (*d*) the GBM pattern combined with radiation.

To examine the correlation of the GBM pattern with an astrocytoma patient’s response to treatment, we classified the 497 patients by chemotherapy or radiation and, in addition, by the GBM pattern (Fig 6). These classifications give bivariate Cox hazard ratios which are close to, and within the 95% confidence intervals of the corresponding univariate ratios (Table A in S1 Appendix). This means that the GBM pattern is a predictor of a patient’s survival independent of treatment, and, therefore, also a predictor of the patient’s response to treatment.

**Fig 7:**
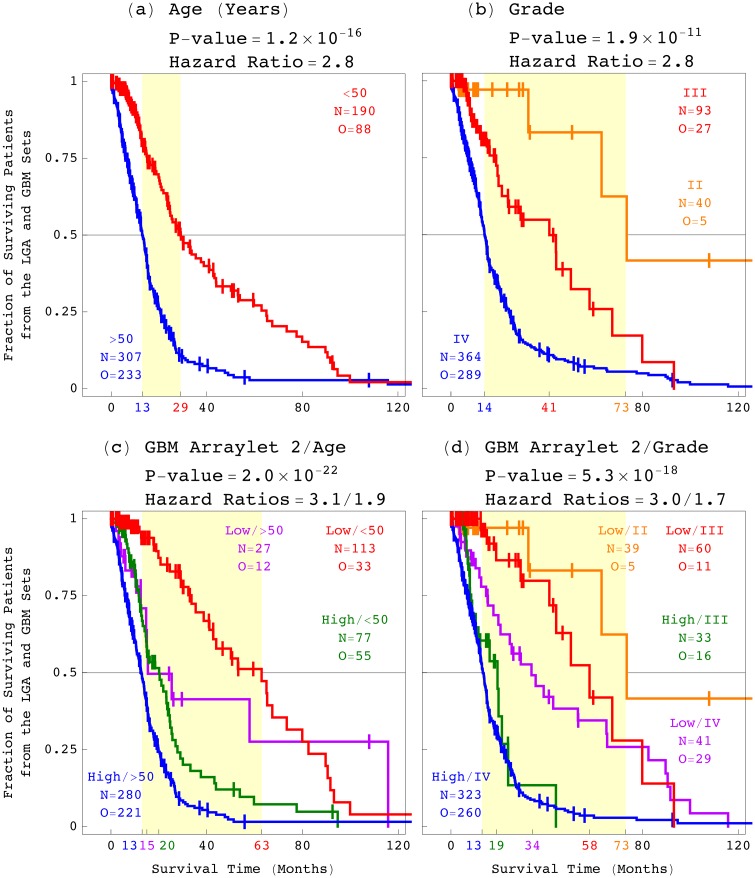
Survival analyses of the astrocytoma patients classified by the patient’s age at diagnosis and the tumor’s grade, and by the GBM pattern. The 497 astrocytoma patients classified by (*a*) the patient’s age, (*b*) the tumor’s grade, (*c*) the GBM pattern combined with age, and (*d*) the GBM pattern combined with grade.

Next, we examined the correlation of the GBM pattern with a patient’s age and a tumor’s grade (Fig 7) [31–38]. We find that the log-rank test *P*-value, which corresponds to the classification by the GBM pattern, is less than the *P*-values which correspond to the classifications by age and grade. The univariate hazard ratio and the concordance index, which correspond to the GBM pattern, are greater than those that correspond to age and grade. These mean that the GBM pattern is statistically a better predictor of astrocytoma outcome than age or grade. Classifying the patients by the GBM pattern in addition to age or grade, we find that the GBM pattern is also statistically independent of age and grade.

Combined with either age or grade, therefore, the GBM pattern is statistically an even better predictor of astrocytoma outcome. For example, the >4-year survival difference among the patients classified by both the GBM pattern and age, is >3 times, and >2.5 years greater than the difference between the patients classified by age alone. The >3.5-year difference among the grades III and IV astrocytoma patients classified by the GBM pattern and grade, is >1.5 times, and 1.5 years greater than the difference between these patients classified by grade alone.

We also compared the GBM pattern to the existing pathology laboratory tests for astrocytoma. Silencing of a tumor’s *MGMT* gene by hypermethylation of its DNA promoter region was associated with a GBM and, recently, also an LGA patient’s longer survival in response to temozolomide chemotherapy treatment [63, 64]. Mutation of the gene *IDH1* was associated with a patient’s longer survival [65], and linked with patterns of mRNA expression and DNA methylation across several hundred genes and genomic regions, respectively, in the tumor’s genome [66–68].

**Fig 8:**
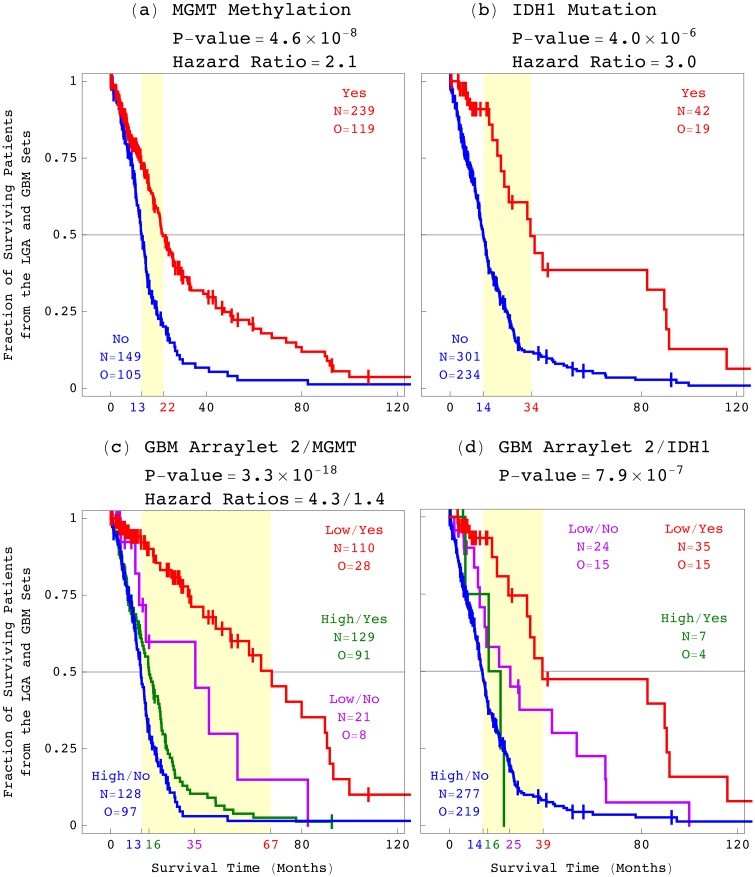
Survival analyses of the astrocytoma patients classified by the existing laboratory tests and by the GBM pattern. The 497 astrocytoma patients classified by (*a*) *MGMT* promoter methylation, (*b*) *IDH1* mutation, (*c*) the GBM pattern combined with *MGMT*, and (*d*) the GBM pattern combined with *IDH1*.

We find that the genome-wide GBM pattern of CNAs is statistically a better predictor of astrocytoma outcome, corresponding to greater median survival time difference, proportional hazard ratio, and concordance index, than *MGMT* promoter methylation and *IDH1* mutation (Fig 8). The GBM pattern additionally classifies the patients with either a methylated or an unmethylated *MGMT* promoter, or a mutated or an unmutated *IDH1*, into two groups each, with an approximately one-year to >4-year survival differences, which means that it is independent of both. Combined with either existing pathology laboratory test, therefore, the GBM pattern is an even better predictor of astrocytoma.

## Discussion

To date, statistically the best indicators of astrocytoma outcome in clinical use remain the patient’s age at diagnosis and the tumor’s grade [31–35, 38]. High-throughput molecular profiling efforts identified two indicative genetic loci that were translated into pathology laboratory tests, one locus of DNA hypermethylation, and the other of DNA mutation linked with mRNA expression and DNA methylation subtypes of astrocytoma [39, 63–68]. Recurring DNA CNAs have been observed in astrocytoma tumors’ genomes for decades, however, copy-number subtypes that are predictive of astrocytoma patients’ outcomes were not identified [36, 37].

Here, we showed that a genome-wide pattern of CNAs in a primary astrocytoma tumor’s DNA copy-number profile is a predictor of the patient’s survival and response to chemotherapy and radiation, statistically better than, and independent of the patient’s age, the tumor’s grade, and the existing laboratory tests. We showed that the pattern is correlated with an approximately one-year survival phenotype among the astrocytoma patients. The pattern is a platform-independent predictor, and, therefore, it can be translated into a laboratory test by using non-disease-specific FDA-approved platforms, such as next-generation sequencing (NGS) [69].

The genome-wide pattern of CNAs was previously uncovered by using the GSVD to model patient-matched copy-number profiles of GBM tumor and normal samples [8]. Here, a GSVD comparison of patient-matched profiles of LGA tumor and normal samples, revealed a tumor-exclusive genome-wide pattern of CNAs. We showed, and computationally validated, that this LGA pattern is correlated with an LGA patient’s outcome. The GSVD separated this pattern from other sources of experimental and biological variation, common to the tumor and normal profiles, or exclusive to the tumor or the normal profiles, without a-priori knowledge of these variations. We also showed that the LGA pattern is encompassed in the GBM pattern, where GBM-specific CNAs encode for enhanced opportunities for transformation and proliferation via growth and developmental signaling pathways in GBM relative to LGA. The LGA datasets had been publicly available in TCGA since 2015, and analyzed by using several methods. The pattern, however, remained unknown until the datasets were modeled by using the GSVD. This illustrates the ability of comparative spectral decompositions in general, and the GSVD in particular to find what other methods miss.

Note that in a GSVD comparison between two datasets, the only assumption is that the structure of the datasets is that of two full column-rank matrices of matched columns. It is, therefore, not limited to profiles of human cells, DNA copy-number profiles, or profiles measured by DNA microarray platforms, nor is it limited to molecular biological datasets. The GSVD was first formulated as a comparative spectral decomposition to model cell cycle phase-matched mRNA expression profiles of synchronized cells from human and yeast [17]. The model predicted a genome-wide causal coordination between DNA replication and mRNA expression [27, 28], which was then experimentally verified [70]. This demonstrated that the GSVD can be used to correctly predict previously unknown cellular mechanisms. Since then, the GSVD has been used to separate the similar from the dissimilar between different species, as well as between different types of molecular biological profiles, mostly large-scale (e.g., mRNA and protein expression in addition to DNA copy-number profiles), and different profiling technologies (e.g., NGS and quantitative real-time PCR in addition to DNA microarray platforms) [18–23] (see also [24–26]).

## Methods

**LGA Discovery Datasets Construction.** We selected an LGA discovery set of 59 TCGA patients of consistent survival annotations. The 59 patients were diagnosed with World Health Organization (WHO) grades III or II astrocytoma. The patient-matched primary LGA tumor and normal tissue samples were obtained from US tissue source sites. Each tumor or normal profile lists median-centered log_2_ TCGA raw level 2 of the Affymetrix Genome-Wide Human SNP Array 6.0-measured DNA copy numbers. The profiles are organized in one tumor and one normal dataset, of *M*_1_, *M*_2_ = 933,827 autosomal and X chromosome nonpolymorphic copy-number probes, with valid data in all *N* = 59 patient-matched pairs of tumor and normal profiles, respectively.

**CNAs in the LGA Pattern.** To compare the Affymetrix-derived LGA pattern to the Agilent-derived GBM pattern, we mapped the 933,827 Affymetrix probes that constitute the LGA pattern onto the National Center for Biotechnology Information (NCBI) human genome sequence build 36 at the University of California at Santa Cruz (UCSC) human genome browser [58]. Previously, we also mapped the 212,696 probes of the Agilent Human Genome CGH 244A microarray platform that constitute the GBM pattern onto the same sequence. We then assigned to the LGA pattern CNAs in the chromosomes and chromosome arms, as well as the 111 of the 130 genomic segments that were previously identified in the GBM pattern by using the circular binary segmentation (CBS) [59], which are of ≥5 Agilent probes in length.

The LGA pattern was assigned a gain or a loss in a chromosome or a chromosome arm if the deviation of the mean copy number of the chromosome or the arm from the genomic mean is greater than twice the genomic standard deviation. The genomic mean and standard deviation are calculated for the autosomal genome, excluding the outlying chromosomes 7 and 10, and chromosome arm 9p [8]. A gain or a loss in a segment were assigned if the deviation of the segment mean copy number from the genomic mean is greater than twice the genomic standard deviation, or if the deviation from the chromosomal mean is greater than the chromosomal standard deviation, when this deviation is consistent with the deviation from the genomic mean.

**Cross-Platform Probe Matching.** We matched pairs of one Agilent and one Affymetrix probe that overlap by at least one nucleotide. When multiple Affymetrix or Agilent probes overlapped a single Agilent or Affymetrix probe, the Affymetrix or Agilent probe closest to the genomic end or start coordinate of the Agilent or Affymetrix probe was selected, respectively, to maintain a one-to-one matching between the platforms. This identified 8,102 pairs of one-to-one overlapping Affymetrix and Agilent probes.

To identify the 4,697 pairs of one-to-one overlapping probes that are consistently aberrated in the LGA and GBM patterns, we assigned to the patterns CNAs in the 8,102 Affymetrix and Agilent probes, respectively. A gain or a loss in a probe were assigned if the deviation of the probe copy number from the genomic mean is greater than twice the genomic standard deviation, or if the deviation from the chromosomal mean is greater than the chromosomal standard deviation, when this deviation is consistent with the deviation from the genomic mean.

**Arraylet Visualization.** To visualize the first tumor arraylet and 53rd normal and tumor arraylets, we segmented each arraylet by using the CBS [59].

**Probelet Interpretation.** To biologically or experimentally interpret the first and 53rd probelets, which are the most significant probelets in the tumor and normal datasets, respectively, we assessed the subsets of patients that are of high or low relative copy numbers in each probelet for enrichment in any one of the multiple TCGA annotations that describe the patients (e.g., gender), and the corresponding tumor and normal tissue samples (e.g., the hybridization plate of the tumor vs. the normal samples). The *P*-value of each enrichment was calculated assuming a hypergeometric probability distribution of the *K* annotations among the *N* patients of the discovery set, and of the subset of *k* ⊆ *K* observed annotations among the subset of *n* patients that are of high or low copy numbers in each probelet [60], $P\left(k;n,N,K\right)={\left(\begin{array}{c} N\\ n \end{array}\right)}^{-1}\overset{n}{\underset{i=k}{\sum }}\left(\begin{array}{c} K\\ i \end{array}\right)\left(\begin{array}{c} N-K\\ n-i \end{array}\right)$.

In each probelet, we also assessed the distribution of the copy numbers among the different groups of each TCGA annotation by using boxplots, and calculating the corresponding Mann-Whitney-Wilcoxon *P*-values.

**LGA Validation Dataset Construction.** We selected an LGA validation set of 74 TCGA patients, which is mutually exclusive of the discovery set. Missing data among the 933,827 Affymetrix probes of the LGA pattern in any of the corresponding tumor profiles were not estimated. The corresponding probes were excluded from the calculations of this profile’s median copy number as well as the profile’s Pearson correlations with the LGA and GBM patterns.

**GBM Dataset Construction.** We selected a GBM set of 364 patients from the previous GBM discovery and validation sets [8]. For patients with more than one primary tumor profile, medians of the profiles were taken. Missing data among the 933,827 Affymetrix probes of the LGA pattern in any of the corresponding tumor profiles were not estimated. The corresponding probes were excluded from the calculations of this profile’s median copy number as well as the profile’s correlations with the GBM pattern.

***MGMT* Promoter Methylation and *IDH1* Mutation Annotations.** To estimate the *MGMT* promoter methylation status of a tumor, we used the TCGA raw level 1 of the Illumina Infinium Human Methylation 27 or 450 BeadChip-measured DNA methylation levels [64].

The *IDH1* mutation status of the LGA and GBM tumors is from TCGA [38, 68].

## Supporting Information

S1 Appendix Figs A–G and Table A. The Mathematica Notebook is available at http://www.alterlab.org/astrocytoma_prognosis/.
(PDF)

S1 Dataset LGA Discovery Set of Patients. The corresponding Affymetrix-measured LGA tumor and normal profiles are at http://www.alterlab.org/astrocytoma_prognosis/.
(TXT)

S2 Dataset GBM Segments. Segments previously identified by the CBS in the GBM pattern [8].
(TXT)

S3 Dataset LGA Segments. Segments identified by the CBS in significant tumor and normal arraylets revealed by the GSVD of the LGA discovery datasets.
(TXT)

S4 Dataset LGA Validation Set of Patients. The corresponding tumor profiles are at http://www.alterlab.org/astrocytoma_prognosis/.
(TXT)

S5 Dataset GBM Set of Patients. The corresponding tumor profiles are at http://www.alterlab.org/astrocytoma_prognosis/.
(TXT)
