# Supplementary material for: Platform-Independent Genome-Wide Pattern of DNA Copy-Number Alterations Predicting Astrocytoma Survival and Response to Treatment Revealed by the GSVD Formulated as a Comparative Spectral Decomposition
Source: PLoS One. 2016 Oct 31;11(10):e0164546. doi: 10.1371/journal.pone.0164546 (PMC5087864; doi:10.1371/journal.pone.0164546)
Supplement: S1 Appendix — The Mathematica Notebook is available at http://www.alterlab.org/astrocytoma_prognosis/. (PDF) [file pone.0164546.s001.pdf]

S1 Appendix: Figs A–G and Table A

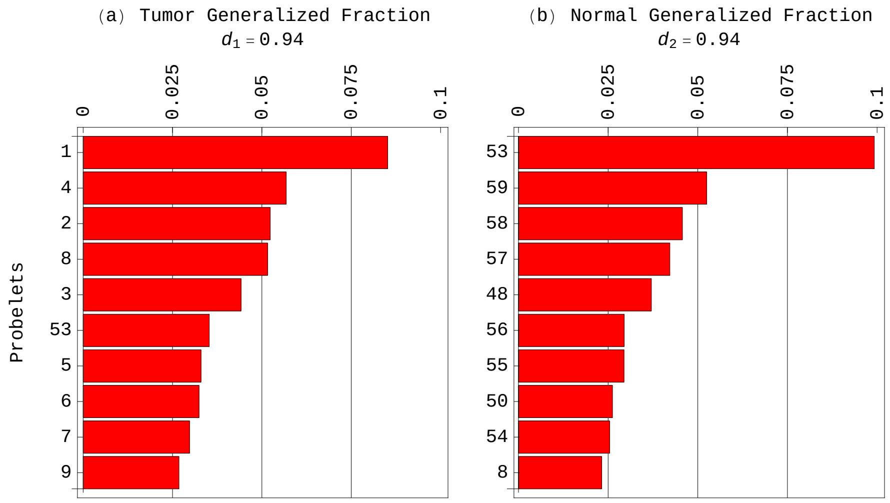

Fig A. Most significant probelets in the LGA discovery, tumor and normal datasets. (*a*) Bar chart of the ten largest generalized fractions of Eq (2) in the LGA tumor dataset shows that the two most tumor-exclusive probelets, i.e., the first and second probelets (Fig 1), are also the first and third most significant probelets in the tumor dataset, with >8% and 5% of the information in this dataset, respectively. The corresponding generalized normalized Shannon entropy of Eq (3) is 0.94. (*b*) Bar chart of the ten largest generalized fractions in the normal dataset shows that the 53rd probelet, which is approximately common to the tumor and normal datasets, is the most significant probelet in the normal dataset with ∼10% of the information.

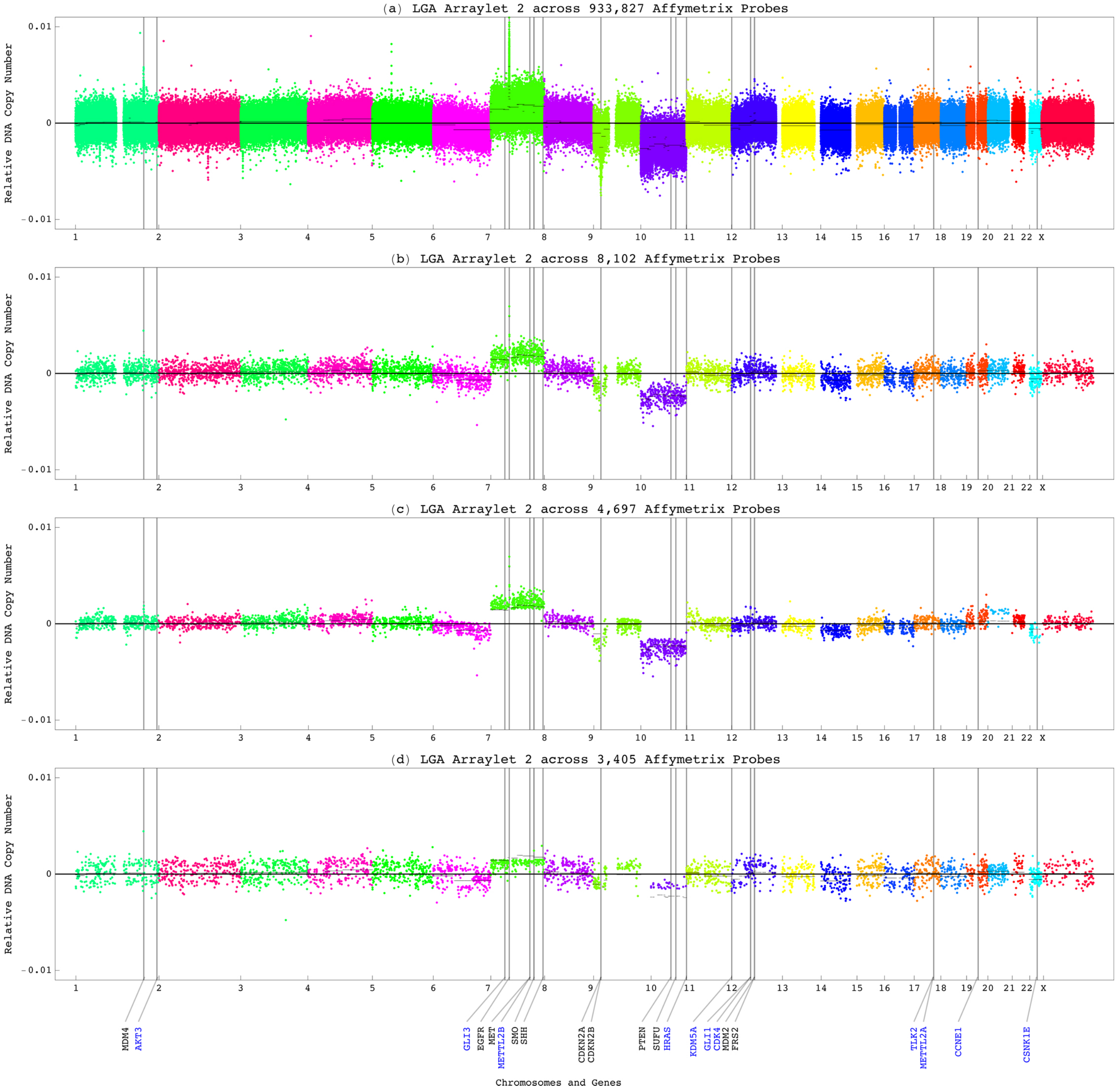

Fig B. LGA genome-wide pattern of co-occurring CNAs revealed by the GSVD of the LGA discovery datasets. (*a*) Plot of the second most LGA tumor-exclusive tumor arraylet, which was revealed by the GSVD, describes a genome-wide pattern of co-occurring CNAs across 933,827 Affymetrix probes. This LGA pattern is encompassed in the GBM pattern (Fig 3), and consists of GBM-associated LGA-shared CNAs (black), including, e.g., gain of a segment on chromosome 1 containing *MDM4*. (*b*) The GBM-associated LGA-shared CNAs, e.g., in *MDM4*, are visible across the 8,102 Agilent-matched Affymetrix probes, even though these are <1% of the probes that constitute the LGA pattern. (*c*) The LGA-shared CNAs are also visible across the 4,697 Agilent-matched consistently-aberrated Affymetrix probes. (*d*) These CNAs are not visible across the 3,405 remaining probes.

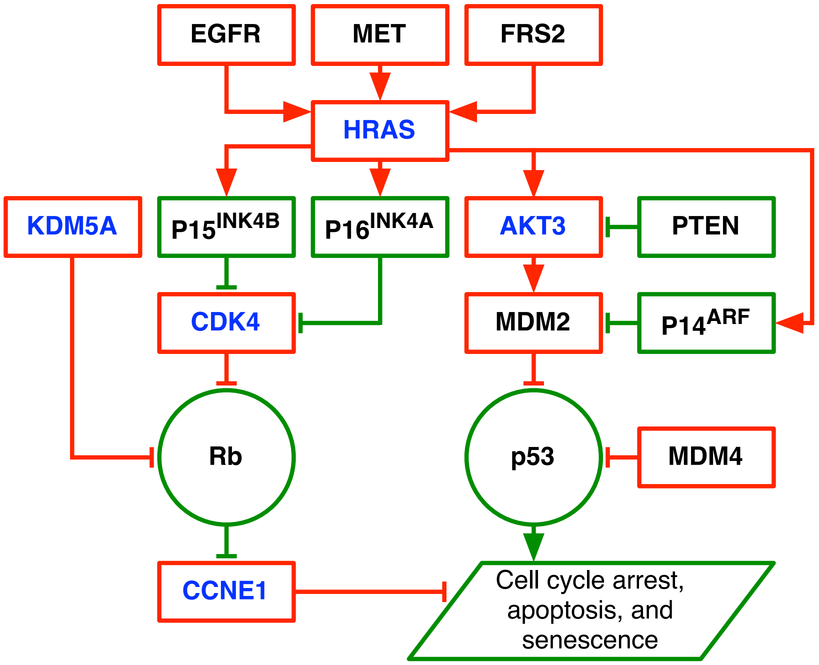

Fig C. GBM-specific CNAs encode for enhanced opportunities for transformation via growth signaling pathways in GBM relative to LGA. Schematic mapping of the GBM and LGA CNAs onto the Ras pathway describes gains (red) and losses (green) of genes and gene transcript variants (rectangles), which are LGA- and GBM-shared (black) or GBM-specific (blue), and relationships, which directly or indirectly lead to increased (lines with arrows) or decreased (lines with bars) activities of the genes and transcripts, and the tumor suppressor proteins p53 and Rb (circles).

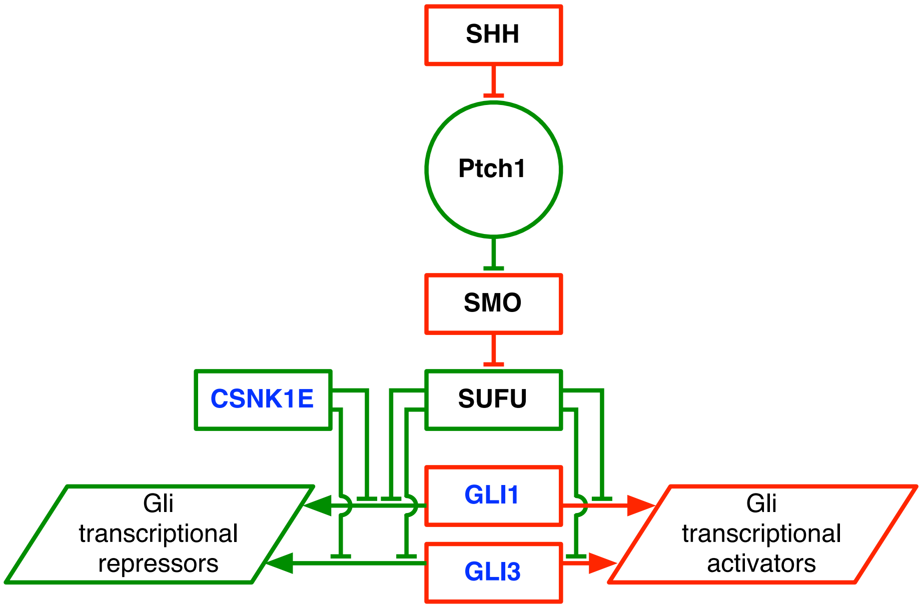

Fig D. GBM-specific CNAs encode for enhanced opportunities for proliferation via developmental signaling pathways in GBM relative to LGA. Schematic mapping of the CNAs onto the Hh pathway describes gains (red) and losses (green) of genes (rectangles), which are LGA- and GBM-shared (black) or GBM-specific (blue), and relationships, which directly or indirectly lead to increased (lines with arrows) or decreased (lines with bars) activities of the genes, and the tumor suppressor protein Ptch1 (circle).

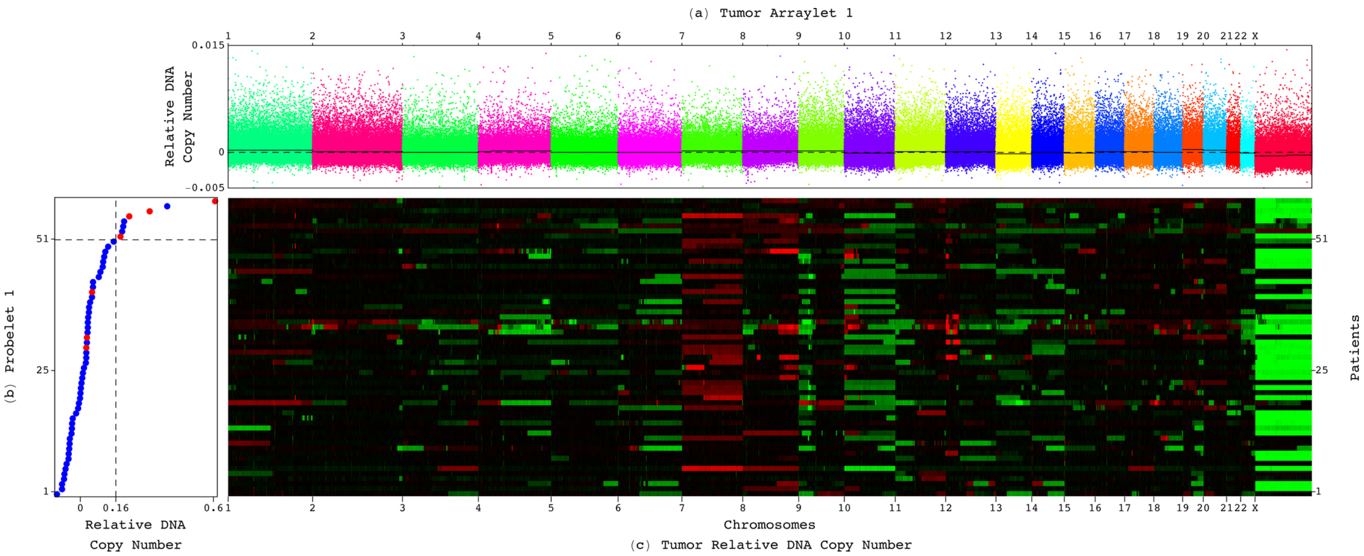

Fig E. The first, most tumor-exclusive probelet and corresponding tumor arraylet revealed by the GSVD of the LGA discovery datasets. (*a*) Plot of the first, most LGA tumor-exclusive tumor arraylet describes mostly unsegmented chromosomes (black lines), each with a copy-number distribution that is approximately centered at the autosomal genome with a relatively large, chromosome-invariant width. The probes are ordered, and their copy numbers are colored, according to each probe's chromosomal location. (*b*) Plot of the first LGA probelet describes the variation of the weight, or superposition coefficient of the first tumor arraylet in the tumor profiles of the 59 patients. The subset of patients that are of high copy numbers is enriched in tumors, which hybridization plate was 2391 (red), rather than other plates (blue). The corresponding hypergeometric P-value is $<10^{-2}$. (*c*) Raster display of the tumor dataset, with relative gain (red), no change (black), and loss (green) of DNA copy numbers, shows the correspondence between the LGA tumor profiles and the first probelet and tumor arraylet, which together describe a tumor-exclusive experimental batch effect.

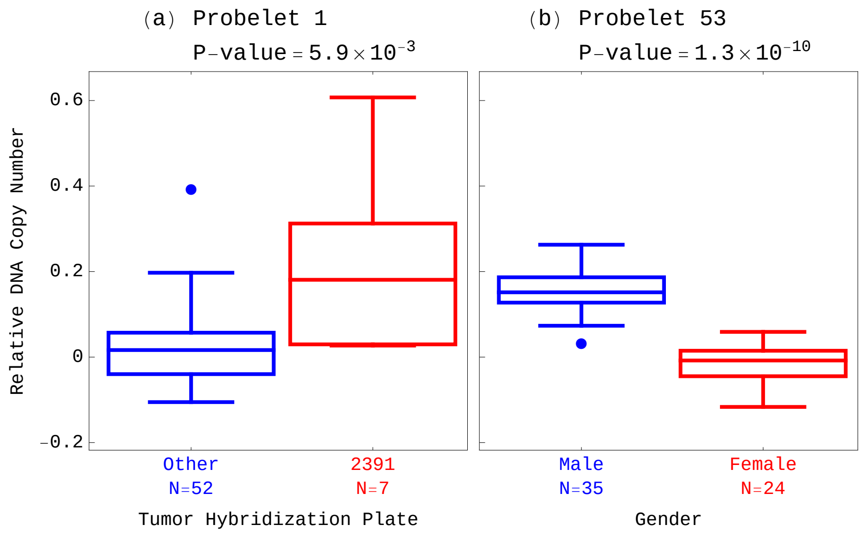

Fig F. Distribution of the copy numbers listed in the significant probelets among the different groups of the TCGA annotations that the probelets are correlated with. (*a*) Boxplots of the distribution of the copy numbers that are listed in the first probelet between two groups of the tumor hybridization plate. The group of tumor hybridization plate 2391 (red) has significantly greater copy numbers than the group of the other plates (blue). The corresponding Mann-Whitney-Wilcoxon P-value is $<10^{-2}$. (*b*) Boxplots of the distribution of the copy numbers that are listed in the 53rd probelet between the two gender groups. The males (blue) have significantly greater copy numbers in the probelet than the females (red), which copy numbers are approximately centered at the autosomal genome. The corresponding Mann-Whitney-Wilcoxon P-value is $<10^{-9}$ (Fig 2).

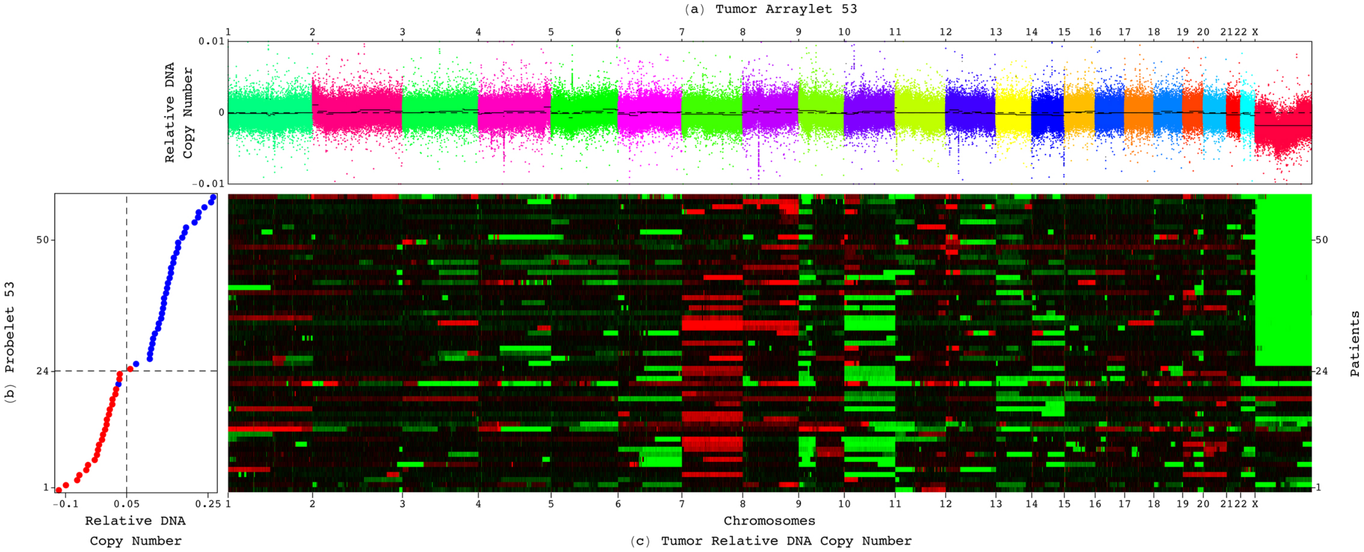

Fig G. The 53rd probelet and corresponding tumor arraylet revealed by the GSVD of the LGA discovery datasets. (*a*) Plot of the 53rd LGA tumor arraylet describes a deletion of the X chromosome. (*b*) Plot of the 53rd LGA probelet, which is approximately common to the tumor and normal datasets, describes a classification of the patients by gender into females (red) and males (blue) (Fig 2). (*c*) Raster display of the tumor dataset shows the male-specific X chromosome deletion across the tumor genomes. This biological variation originated in the patient-matched LGA normal genomes. The GSVD separates this variation from the second LGA tumor arraylet.

Cox Proportional Hazard Model	Predictor	Hazard Ratio	95% Confidence Interval	P-value	Concordance Index
Univariate	GBM Arraylet 2	4.1	3.0–5.8	2.9×10^{-17}	0.85
	Chemotherapy	1.9	1.5–2.5	4.7×10^{-7}	0.72
	Radiation	2.6	2.0–3.4	3.4×10^{-13}	0.80
	Age	2.8	2.2–3.6	1.4×10^{-15}	0.80
	Grade	2.8	2.0–3.7	1.4×10^{-10}	0.82
	MGMT	2.1	1.6–2.7	8.6×10^{-8}	0.66
	IDH1	3.0	1.8–4.9	1.0×10^{-5}	0.83
Bivariate	GBM Arraylet 2	4.5	3.2–6.3	6.1×10^{-19}	0.81
	Chemotherapy	2.2	1.7–2.8	6.3×10^{-10}	
	GBM Arraylet 2	4.5	3.2–6.2	7.3×10^{-19}	0.84
	Radiation	3.0	2.3–3.9	3.1×10^{-16}	
	GBM Arraylet 2	3.1	2.2–4.4	5.7×10^{-10}	0.79
	Age	1.9	1.4–2.5	7.5×10^{-6}	
	GBM Arraylet 2	3.0	2.1–4.3	4.2×10^{-9}	0.80
	Grade	1.7	1.2–2.4	1.5×10^{-3}	
	GBM Arraylet 2	4.3	2.9–6.3	2.4×10^{-9}	0.73
	MGMT	1.4	1.1–1.9	1.0×10^{-2}	

Table A. Cox proportional hazard models of the 497 astrocytoma patients classified by the GBM pattern, treatment, age and grade, and existing laboratory tests.
